# Using Platelet-Rich Fibrin to Remove Graphite Tattoos May Yield Excellent Long-Term Result

**DOI:** 10.1155/2024/5559986

**Published:** 2024-06-24

**Authors:** Young Joon Cho, Yong Tak Jeong, Tae Hee Lee, Hyun Woo Cho

**Affiliations:** ^1^ Mac Dental Clinic, Daegu, Republic of Korea; ^2^ Department of Periodontics School of Dentistry Kyungpook National University, Daegu, Republic of Korea; ^3^ New York University College of Dentistry New York University, New York, USA

## Abstract

Graphite tattoos are rarely reported because they are mainly caused by an accidental injury or habits during childhood that cause a pencil to penetrate the oral mucosa. Unlike other pigmentations, it stains layers that are deeper than the subepithelial and mucosal layers, and in most cases, it takes the form of a grayish black macule. This case report describes depigmentation with the denudation technique that was followed by a novel approach of using platelet-rich fibrin to cover exposed bone. A 41-year-old male patient presented with an aesthetic complaint from a grayish black staining on the labial gingiva near the maxillary central and lateral incisors. The lesion was diagnosed as a graphite tattoo due to the patient's history of sticking his gum with pencils when he was young. The entire pigmented gingiva was surgically removed and covered with two layers of PRF membrane to protect the exposed bone surface and provide an extracellular matrix for migration of gingival fibroblasts. Healing patterns were observed at 1, 2, 4, and 8 weeks, and satisfactory clinical and aesthetic results were obtained. Creeping attachment was observed at 8 years postop, and there was no recurrence for a long-term period of 13 years.

## 1. Introduction

Gingival pigmentation of the maxillary anterior teeth causes a patient to take a passive attitude when smiling or talking to others and can also cause psychological withdrawal. Pigmentation of soft tissues in the oral cavity can be classified into pathologic, physiologic, and nonphysiologic depending on the development process, and it can also be divided into endogenous and exogenous, depending on the origin of the pigment causing the staining [1, 2]. Melanin pigmentation, which is of endogenous origin produced by melanocytes, is the most common staining in oral soft tissues. Amalgam tattoo and graphite tattoo are categorized as nonphysiologic exogenous pigmentation. Graphite tattoos are rarely reported but are thought to be more common within the population. Unlike other pigmentations, they stain deeper than the subepithelial or mucosal layer, and in most cases, they take the form of a grayish black macule. They are clinically similar to an amalgam tattoo, but the two lesions can be distinguished radiologically, historically, and histologically [3, 4]. Amalgam tattoos are visible on radiographs when amalgam particles are large, but graphite tattoos are not visible regardless of particle size. While amalgam tattoos are associated with a history of amalgam restoration or removal, graphite tattoos are mostly associated with a history of trauma, mainly related to stabbing pencils during childhood [3, 5–7].

Although various treatments for depigmentation have been introduced, there has not been a therapy that yields satisfactory results. Treatment methods introduced so far are surgical methods, cryosurgery, laser, electrosurgery, and chemical methods, and dentists usually cover the depigmented soft tissue with a free gingival graft (FGG), a connective tissue graft (CTG), or commercial products like acellular dermal matrix [8].

Graphite tattoo is mainly caused by carbon particles penetrating close to the bone. Thus, a denudation technique is the most effective therapy, as it removes the entire pigmented gingiva. For this technique, if the exposed bone surface is not protected after gingival removal, complications such as pain, discomfort, prolonged healing, recession of adjacent teeth, and marginal bone loss may occur during secondary intention wound healing [9]. This necessitates bone protection after a push-back method. FGG and CTG, which are used to cover the exposed bone surface, are technique sensitive, require an additional surgical field for donor site, and have limited amount of harvesting. If dentists choose to use commercial products like acellular dermal matrix in order to avoid secondary trauma to the donor site, additional costs are generated while the results are inferior to autogenous grafting.

Platelet-rich fibrin (PRF), a second-generation platelet concentrate, has been in the spotlight as a wound dressing or a matrix that promotes healing [10]. Recent research has revealed that PRF has anti-inflammatory effects and increases cell proliferation, migration, and differentiation during wound healing [11]. To our knowledge, despite these many advantages of PRF regarding wound healing, there has been no report on the use of PRF for the procedure of depigmentation. Therefore, this case report is aimed at reporting a long-term prognosis after using PRF to cover exposed bone after the denudation technique for removal of a graphite tattoo.

## 2. Case Report

**Figure 1:**
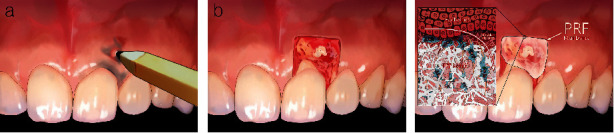
(a) Graphite tattoo was caused by sticking gum with pencil. (b) Push-back method, which removes all soft tissue above alveolar bone, was established as treatment planning in order to prevent recurrence. (c) PRF membrane over exposed alveolar bone can provide extracellular matrix and growth factors to facilitate migration of gingival fibroblasts from adjacent keratinized gingiva.

**Figure 2:**
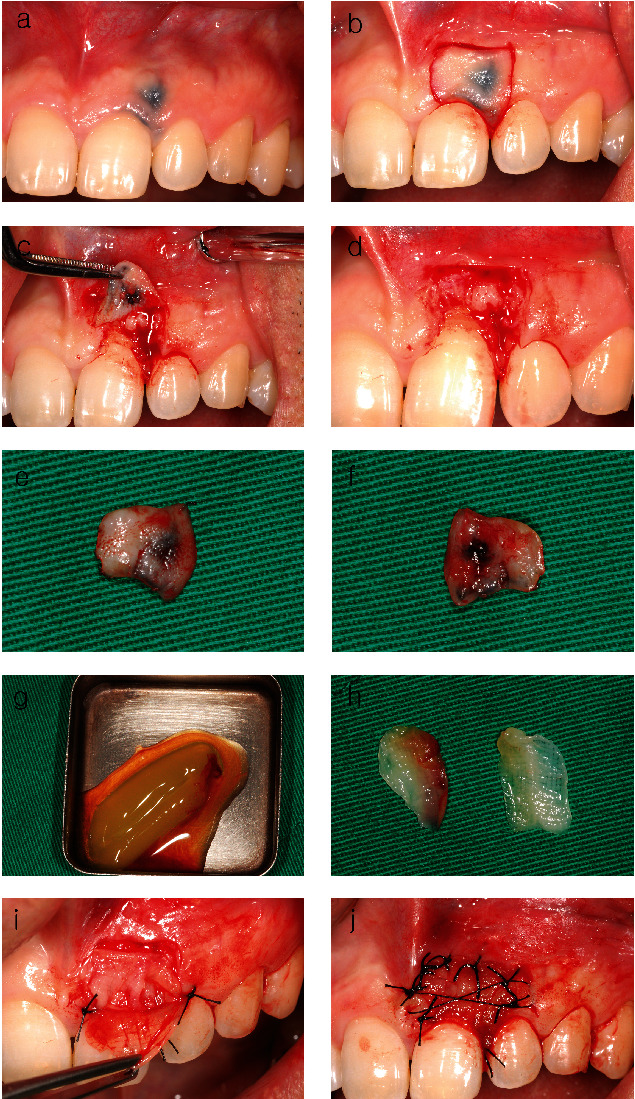
(a) Graphite tattoo extending to the interdental papilla and marginal gingiva of the maxillary left central incisor and lateral incisor. (b) Incision design to remove the graphite tattoo and surrounding gingival tissue. (c) Excisional procedure with a full thickness flap. (d) Hidden root recession and traumatic bone injury caused by the patient's habit of sticking his gum with a pencil. (e) Excised gingival tissue including the graphite tattoo. (f) Inner surface of excisional gingiva. The graphite tattoo approached the periosteum. (g) PRF after centrifuge. (h) PRF membranes formed by squeezing the PRF clot with a couple of 2 × 2 gauze. (i) Positioning the inner PRF membrane and suturing the outer PRF membrane with 5-0 braded black silk (Ailee Co. Ltd.). (j) Fixation of PRF membranes on alveolar bone with direct loop and cross mattress sutures.

The case report, approved by the Public Institutional Review Board appointed by the Ministry of Health and Welfare (P01-202310-01-040), incorporates the patient's submission of a legally binding consent form. A 41-year-old male patient presented with an aesthetic complaint from soft tissue pigmentation on the maxillary anterior region. Regarding past medical history, he had never been diagnosed with any particular disease and had never taken any significant medications. He was a nonsmoker. No other pathological findings other than the gingival tattoo were observed. He had a habit of sticking his gum with a pencil when he was young (Figure 1(a)). Upon clinical observation, grayish black macules were observed, extending from the interdental papilla and marginal gingiva to the apex of the maxillary left central incisor and lateral incisor (Figure 2(a)). We diagnosed the lesion as a graphite tattoo. In order to prevent recurrence, we planned to use the push-back method to remove the entire mucosa above the alveolar bone (Figure 1(b)) and then to cover the exposed alveolar bone with a PRF membrane to induce migration of gingival fibroblasts from adjacent keratinized gingiva (Figure 1(c)).

## 3. Material and Methods

### 3.1. Surgical Procedure

Mouthwash was performed with 0.12% chlorhexidine digluconate (Hexamedine solution 250 mg, Bukwang Pharma Co.). Local anesthesia (2% lidocaine with 1 : 80,000 epinephrine, Yuhan Pharm. Co.) was injected into the buccal gingiva and vestibule to anesthetize the anterior superior alveolar nerve. Excisional procedure with a #15 blade was performed to remove the pigmented gingiva and surrounding soft tissues, and the size of removed tissue was 1 × 1 cm (Figures 2(b), 2(c), and 2(d)). When we removed the graphite tattoo, we observed hidden root recession and traces of traumatic bone injury, suspected to have been caused by the patient's habit of using a pencil to stab the gingiva (Figure 2(d)). As expected, the graphite tattoo approached the periosteum above the alveolar crest (Figure 2(f)). Unfortunately, histological examination of the collected specimens could not be performed. 10 cc of venous blood was collected and centrifuged with 400 g force for 10 minutes to make PRF [12]. We chose not to use a commercial kit; instead, we prepared PRF membranes by compressing the PRF clot (1 × 3 cm in size) with a couple of 2 × 2 gauze (Figures 2(g) and 2(h)). First, a sheet of PRF membrane was placed above the alveolar bone to serve as an extracellular matrix for migration of gingival fibroblasts from adjacent keratinized gingiva. Another sheet of PRF membranes was covered to protect the inner PRF membrane. Then, PRF membranes were sutured with direct loop sutures and a cross mattress suture (Figures 2(i) and 2(j)). The patient received a prescription for naproxen 500 mg twice daily as an analgesic and anti-inflammatory medication, alongside a mouth rinse containing 0.2% chlorhexidine, both to be used for a three-day duration. Removal of the stitches occurred on the fourth day to minimize interference with the healing process caused by the sutures. Despite the absence of complications or delays in wound healing, it is regrettable that we could not assess patient-reported outcomes such as the visual analogue scales, verbal descriptor scale, or verbal rating scale [13].

## 4. Results

**Figure 3:**
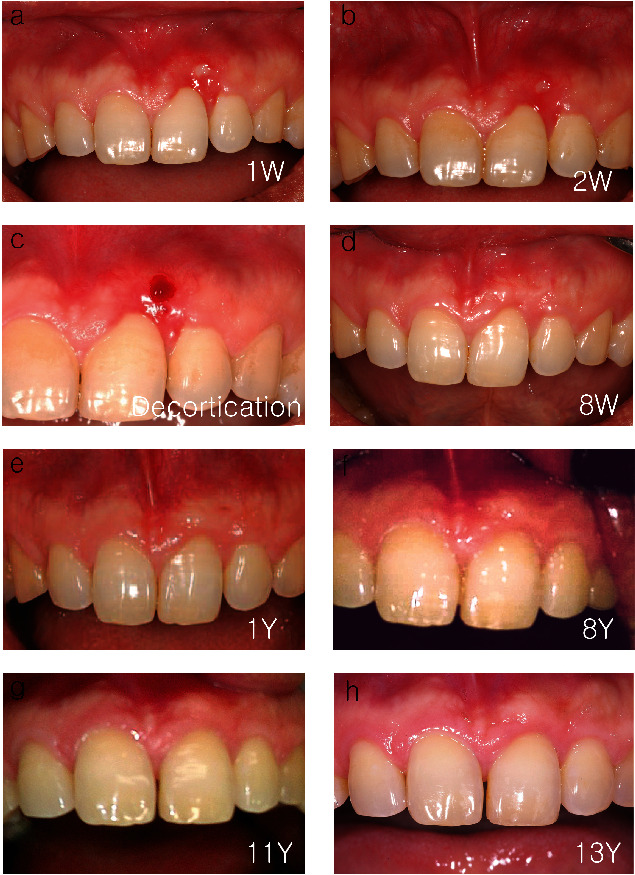
(a) At 1 week, overall soft tissue covering was achieved. But small amount of bone exposure was still observed. (b) At 2 weeks, the size of the exposed bone was reduced, but exposure still remained. (c) Bleeding was induced by a #8 round carbide bur for soft tissue regeneration. (d) At 4 weeks, exposed bone was completely covered with regenerated soft tissue, and soft tissue healing was almost completed, with a small amount of root exposure. (e) Clinical photo at 1 year. (f) At 8 years, creeping attachment was observed. (g) Clinical photo at 11 years. (h) Clinical photo at 13 years.

After performing the denudation technique, clinical photographs were taken throughout the 13 years of follow-up period (Figure 3). No significant complications occurred during the healing process. Based on the patient's account, discomfort and pain during everyday activities like chewing and speaking persisted for about three-day postsurgery. Yet, these sensations were effectively managed with the prescribed analgesic and anti-inflammatory medication, eliminating the need for additional pain relief. By the seventh day following the procedure, discomfort had significantly diminished, with no noteworthy pain or discomfort reported thereafter.

When we removed the graphite tattoo, we found hidden gingival recession and a small area of exposed bone in the area, and both were suspected to have been caused by traumatic injury associated with the patient's history of sticking pencils in his gingiva (Figures 3(a) and 3(b)). At 2 weeks, bleeding was induced by drilling with a #8 round carbide bur to stimulate soft tissue regeneration from the bone marrow (Figure 3(c)). At 4 weeks, regenerated gingiva covered exposed bone. At 8 weeks, although there was some root exposure, overall healing was completed (Figure 3(d)). Despite our inability to evaluate patient-reported outcome measures using official scales [13], the patient expressed high aesthetic satisfaction and did not report any discomfort or pain. Although there was no significant difference during the first year (Figure 3(e)), creeping attachment was observed at 8 years (Figure 3(f)). The patient was satisfied with the results of the depigmentation therapy as its results were well maintained without recurrence for 13 years (Figures 3(g) and 3(h)).

## 5. Discussion

Unlike physiologic pigmentation in the oral cavity, graphite tattoo often penetrates deep into the mucous membrane and sometimes beyond the periosteum. Therefore, there are many cases in which the periosteum and sometimes even some bone tissue must be removed to achieve complete depigmentation. As a result, clinicians are often left with no choice but to have the surgical area heal by secondary intention without any gingival connective tissue while leaving the bone surface exposed. In such cases, epithelial migration on bone can only be initiated by granulation tissue because there is no gingival connective tissue [14]. Experiments on monkeys showed that placing a soft tissue graft on bone resulted in retardation of initial healing during the first 2 weeks, when compared to placing a soft tissue graft on periosteum [15]. To address this issue, in this case report, PRF was used to cover the exposed bone surface, and long-term results were satisfactory (Figure 3). Currently, PRF is being used as an autogenous matrix to promote healing of soft tissue and hard tissue. In shallow wounds, epithelial cells can migrate directly through the fibrin clot, obviating the need for the presence of gingival connective tissue [16]. Fibrin clot, which is the first to form at the wound when soft tissue is damaged, acts as a reservoir of growth factors and cytokines and as a provisional substrate for the migration of monocytes and fibroblasts [17]. At the one-week clinical assessment, it was noted that the healing progress seemed to be more rapid compared to conventional denudation procedures or soft tissue grafts, a phenomenon attributed to the efficacy of PRF. This corresponds with the results reported by Yuan et al. in 2024, which demonstrated that the use of PRF reduced pain and promoted healing [18]. Hence, it is inferred that utilizing PRF for depigmentation procedures may offer greater benefits compared to methods such as simple denudation procedures or the use of FGG or CTG. FGG and CTG are surgical methods widely used to protect the surgical site after tissue removal. However, the need for secondary healing at the donor site to obtain autografts can cause increased discomfort for patients during and after surgery. Furthermore, FGG may present challenges such as color and texture mismatches in soft tissue, especially in aesthetically sensitive areas like the maxillary anterior region, after healing.

The recurrence rate of surgical treatments for deepithelialization, such as abrasion using a diamond bur, laser, or scalpel—which is mainly performed to remove oral melanin pigmentation—is relatively high [19, 20]. This is thought to be caused by failure to completely remove not only the pigmented area but also melanocytes that reside in deeper layers of epithelium. Therefore, the push-back method, which completely removes soft tissue above alveolar bone, is effective at preventing recurrence [21]. However, if the exposed bone surface is left as is, side effects such as gingival recession, alveolar bone resorption, and delayed healing are likely to occur. FGG and CTG, which are used to protect the surgical area after depigmentation, require an additional surgery causing secondary wounds and patient's discomfort from preparing the donor site, and the amount of graft that can be obtained is limited [21]. If a commercial product such as acellular dermal matrix is used, preparation of a donor site is not necessary. However, additional costs will be incurred, and it also introduces a risk of host immune reaction against the foreign material. Therefore, the use of PRF to cover exposed bone after the denudation technique has a unique advantage of reducing time required for surgery and the patient's discomfort caused by secondary wounds. The properties of the epithelium are mainly determined by the underlying connective tissue [22], and in this case, it is presumed that regeneration of the attached gingiva is caused by fibroblasts migrating from the supra-alveolar connective tissue, keratinized gingiva adjacent to incision line, and periodontal ligament to the PRF membrane. Among these tissues, periodontal ligament in particular plays an important role. When surrounding keratinized tissue is removed from teeth entirely, new keratinized tissue regenerates after a certain healing period [22, 23]. However, when keratinized mucosa around an implant is completely excised, the same phenomenon does not occur [23]. For this reason, periodontal ligament is considered to be crucial for the formation of new keratinized tissue. In the context of this particular case report, all keratinized tissue in the discolored area was removed to eliminate a graphite tattoo that had penetrated the periosteum. However, over time, complete reconstruction with new keratinized tissue was observed, and it is assumed that the healthy periodontal ligament surrounding the tooth was what drove the regeneration.

Creeping attachment was observed at 8 years (Figure 3(f)). Up until now, creeping attachment has been consistently reported after grafting for root coverage [24], but it has never been reported after using PRF. Although the purpose of using PRF in this case report was to protect the bone exposed after denudation technique, we found that PRF may also be effective in root coverage for hidden gingival recession and creeping attachment (Figures 2(d) and 3(f)). Further research is needed to confirm the effects of PRF when it comes to covering the exposed connective tissue or bone surface.

Unfortunately, we were not able to include a histologic assessment of the removed lesion. However, biopsy results of graphite tattoos are already included in many reports, and their histologic characteristics are already well established. Furthermore, it is the thorough review of the patient's history that can identify the specific source of extrinsic pigmentations—in this case, a pencil. Overall, our novel approach at removing graphite tattoos was able to yield successful long-term results.

## 6. Conclusions

When removing pigmented soft tissue in the oral cavity, such as graphite tattoos, using PRF after the denudation technique can be a simple and cost-effective treatment option. PRF can be used to cover exposed bone and therefore has a unique advantage of reducing surgical time and the patient's discomfort, which is not seen in any other technique for removing pigmented soft tissues. Surgical results using PRF can also be more reliable compared to using an acellular dermal matrix, as PRF is an autogenous material. In this case report, combining PRF and the denudation technique provided excellent aesthetic results under long-term observation. Although our report lacks a histological assessment, the patient's history confirms the diagnosis of graphite tattoo.

In conclusion, using PRF after the denudation technique is considered to be an effective procedure and prevents repigmentation in long-term prognosis. Further research is needed to verify the effects of PRF regarding its ability to promote healing over exposed surfaces of bone.

## Data Availability

All clinical photos and graphic drawings are included in this manuscript.
